# Efficacy and Safety of Fingolimod in an Unselected Patient Population

**DOI:** 10.1371/journal.pone.0146190

**Published:** 2016-01-06

**Authors:** Maria Rasenack, Jonathan Rychen, Michaela Andelova, Yvonne Naegelin, Christoph Stippich, Ludwig Kappos, Raija L. P. Lindberg, Till Sprenger, Tobias Derfuss

**Affiliations:** 1 Department of Neurology, University Hospital Basel, Basel, Switzerland; 2 Department of Biomedicine, University Hospital Basel, Basel, Switzerland; 3 Department of Radiology, Division of Neuroradiology, Basel, Switzerland; 4 Department of Neurology, DKD Helios Klinik Wiesbaden, Wiesbaden, Germany; University of Düsseldorf, GERMANY

## Abstract

**Background:**

Fingolimod is a first in class oral compound approved for the treatment of relapsing-remitting multiple sclerosis (RR-MS). The aim of this study was to evaluate clinical and neuroradiological responses to fingolimod as well as the safety and tolerability in RR-MS patients in clinical practice. In addition, a panel of pro-inflammatory serum cytokines was explored as potential biomarker for treatment response.

**Methods:**

We conducted a retrospective, non-randomized, open-label, observational study in 105 patients with RR-MS and measured cytokines in longitudinal serum samples.

**Results:**

Compared to the year before fingolimod start the annualized relapse rate was reduced by 44%. Also, the percentage of patients with a worsening of the EDSS decreased. Accordingly, the fraction of patients with no evidence of disease activity (no relapse, stable EDSS, no new active lesions in MRI) increased from 11% to 38%. The efficacy and safety were comparable between highly active patients or patients with relevant comorbidities and our general patient population.

**Conclusions:**

The efficacy in reducing relapses was comparable to that observed in the phase III trials. In our cohort fingolimod was safe and efficacious irrespective of comorbidities and previous treatment.

## Introduction

Fingolimod has been approved as first oral agent for the treatment of relapsing-remitting MS (RR-MS). It is a sphingosine 1-phosphate (S1P)-receptor modulator which binds to the S1P-receptors and induces their downregulation on the cell surface [1]. This downregulation leads to a sequestration of lymphocytes in lymphnodes which has been proposed to be the major mode of action.

A comprehensive clinical program has shown the efficacy and safety of the drug [2, 3]. Fingolimod treatment led to a more than 50% reduction in relapse rates compared to placebo or a standard interferon treatment [2, 3]. Data on disability progression also favored fingolimod treated patients. These clinical results were corroborated by MRI data.

Regarding side effects the impact of fingolimod on cardiac conduction and its effect on the immune response raised some concern. Since patient groups with certain cardiac risk factors were excluded from pivotal trials, the safety and tolerability of fingolimod in these patient groups is less studied. It is obvious that a different patient population is treated in clinical practice compared to clinical trials, which may influence tolerability and efficacy of the drug. It is therefore important to assess these parameters of fingolimod in a cohort of patients in routine clinical practice. We followed a cohort of 105 patients in our center for one year and recorded clinical and MRI efficacy parameters as well as side effects. Biomarkers to assess disease evolution and treatment response are urgently needed in MS. Serum cytokines were shown to be significantly altered in MS patients [4]. Due to their accessibility serum cytokines would be an attractive biomarker. We hypothesized that the fingolimod-induced dramatic reduction of lymphocytes in blood leads to an alteration of cytokines that could be correlated to clinical outcomes and studied this in an exploratory analysis.

## Material and Methods

### Ethics Statement

This retrospective study was approved by the local ethics committee EKBB (Ethikkommission beider Basel 49/06 and master thesis ethics committee). Patient records and information was anonymized and de-identified prior to analysis. The subgroup of patients that had blood samples taken for cytokine analysis signed an informed consent form. 105 RR-MS patients were included in the study. They started treatment with fingolimod (0.5 mg daily) between 04/2011 and 03/2012 and were followed up for one year. Patients who stopped fingolimod treatment during this one year follow-up were included in the overall analysis where data were available. Baseline parameters included patient demographic information and medical history. Cardiac parameters (ECG, heart rate, blood pressure) were analyzed for six hours during first dose monitoring. PQ intervals longer than 200 ms and QTc intervals longer than 450 ms were regarded as prolonged. Standardized neurological examination using the EDSS and relapse assessment were recorded one year before start of fingolimod, at baseline and one year after treatment start. The EDSS was not reevaluated after the end of the study. Routine lab parameters were controlled at baseline, 3 weeks, 3 months, 6 months, and one year after treatment start. Where available, T2 lesion count and gadolinium enhancing lesions were analyzed in a pretreatment MRI, a baseline MRI at treatment start and a follow-up MRI after one year of therapy.

An additional blood draw at baseline and after 3 months of therapy was taken in a subset of 33 patients. In the sera of these patients granulocyte/macrophage colony-stimulating factor (GM-CSF), interferon (IFN)-gamma, tumor necrosis factor (TNF)-a, interleukin (IL)-1b, IL-2, IL-6, IL-8, IL-10, and IL-12p70 were quantified by the MSD HumanUltra-Sensitive Pro-inflammatory 9-plex electrochemiluminescent (ECL) assay (MSD: Meso Scale Discovery, Gaithersburg, MD) according to the manufacturer’s guidelines. Samples were analyzed in duplicate and the plates were read in a SECTOR Imager instrument (Meso Scale Discovery). The longitudinal evolution of cytokines was correlated with clinical and MRI parameters to look for potential predictive biomarkers, the results were compared using the Mann-Whitney U-test. Since 9 cytokines were analyzed we corrected for multiple testing with the Bonferroni correction. Statistical analysis was done using the Student’s paired t-test for routine lab parameters, cardiac parameters durinf first dose monitoring, MRI parameters and the comparison between patients formerly under highly active pretreatment and treatment naïve patients. The annualized relapse rate in the year before and during the first year of Fingolimod treatment was analyzed by the Wilcoxon test. The correlation between ARR and lymphocyte counts was calculated using the Pearson correlation test. The evolution of the EDSS with the percentages of patients that improved, remained stable or worsened during the observation period and the proportion of patients with no evidence of disease activity (NEDA) were compared using the chi square test.

## Results

A cohort of 105 relapsing-remitting MS patients who newly started on fingolimod was followed for one year. Baseline characteristics of these patients are shown in Table 1.

**Table 1:** Baseline characteristics of our cohort compared to the FREEDOMS cohort.

	our cohort(n = 105)	FREEDOMS(n = 425)
Age–year/ mean	44.7	36.6
range	24–65	18–55
Female sex %	62	69.6
Time from first MS symptoms to start of therapy–years/ mean	13.4	8.0
range	1.5–35	0–35
Number of relapses within previous year/ mean	0.67	1.5
range	0–2	0–5
EDSS score/ mean	3.1	2.3
range	0–7	0–5.5
No history of disease modifying treatment—%	12	57.4

Although fingolimod is a first line treatment in Switzerland, 88% of patients were pretreated with other disease modifying drugs. 38% of these had more than one pretreatment. 19% were de-escalated from natalizumab, mitoxantrone or rituximab. The wash-out period for patients previously treated with natalizumab was between 2 and 3 months. Patients formerly treated with mitoxantrone and rituximab had a wash-out period of 6 months. There was no wash-out phase in patients who had a pre-treatment with glatirameracetate or interferons. Relevant concomitant diseases included coronary heart disease in 3%, arterial hypertension in 4.7%, diabetes mellitus in 4%, recurrent uveitis in 1%, chronic obstructive pulmonary disease (COPD) in 2% and bronchial asthma in 1% of patients. 19 patients were treated with a medication potentially prolonging the QTc-time.

During first dose monitoring 64% of patients showed an increased PQ-interval (mean prolongation: 11ms; p< 0.01) and 48% showed a longer QTc time (mean prolongation: 2 ms; p = 0.29) compared to before treatment. An AV-block grade I developed in 4% of patients and grade II Mobitz type 2 in 1% of patients. The AV-blocks resolved within 24 hours. Concomitant medication potentially prolonging the QTc time (Table 2) increased the QTc time with a mean prolongation of 6 ms (p = 0.3) and the PQ time with a mean prolongation of 15 ms (p = 0.36) as compared to no such medication. Only one of these patients (treated with Tizanidin) developed an AV-block. There were no symptomatic cardiac events. None of the patients returned to the hospital due to cardiac complaints during follow-up.

**Table 2:** Concomitant medication that potentially prolongs the QTc time and number of patients taking the respective treatment.

Concomitant medication	no of patients
Tolterodine	1
Tizanidine	5
Paroxetine	1
Quetiapine	1
Citalopram	4
Alfuzosine	1
Trimipramine	2
Olanzapine	1
Metoprolol	1

The differential blood cell count showed a significant drop in lymphocyte levels of approximately 73% after 3 weeks of treatment (p<0.01). 11 patients showed a decrease of lymphocytes below 200 x10^6^/l at least one time during the one year observation period. They did not report an increased infection rate. There was no difference regarding immunosuppressive pretreatment between the patients with pronounced lymphopenia and the whole cohort (p = 0.23).

The adverse effects most often reported by the patients are shown in Table 3. Of note herpes zoster occurred in 3% of patients 2–7 months after start of fingolimod. All these patients were VZV seropositive and at the time of presentation with herpes zoster, lymphocyte levels were well above 200 x10^6^/l and granulocyte levels were in the normal range. One of these patients was pretreated with multiple immunosuppressants, the others received only immunomodulatory pretreatments.

**Table 3:** Adverse effects during the first year of fingolimod treatment; a given patient could name several adverse effects.

Adverse effects	no of patients
Fatigue	8
Increased infections	5
Herpes zoster	3
Headache	3
Herpes labialis	2
Oral aphthae	2
Dizziness	2
Nausea, stomach ache, diarrhea	2
Prolonged infections	1
Increased sweating	1
Muscle ache	1
Weight loss	1
Dyspnea	1
Macula edema	1

Overall fingolimod treatment was stopped in 6% and temporarily paused in 10% of our patients. The reasons for treatment stop or pause are shown in Tables 4 and 5. In the majority of patients in which fingolimod treatment was suspended the treatment pause lasted between 9 and 12 days. Only 2 patients had a treatment pause exceeding 14 days (21 days and 4 months respectively).

**Table 4:** Adverse effects leading to a stop of fingolimod therapy, n = 6

Stop of therapy no.	6
reasons:	
High relapse rate	2
Macula edema	1
Dyspnea	1
Chemotherapy	1
psychological disturbances	1

**Table 5:** Adverse effects leading to a pause of fingolimod therapy, n = 10.

Pause of therapy no.	10
reasons:	
Infections	3
Herpes Zoster	2
Elevated liver enzymes	2
Pronounced lymphocytopenia	1
Pregnancy	1
Dyspnea	1

**Fig 1:**
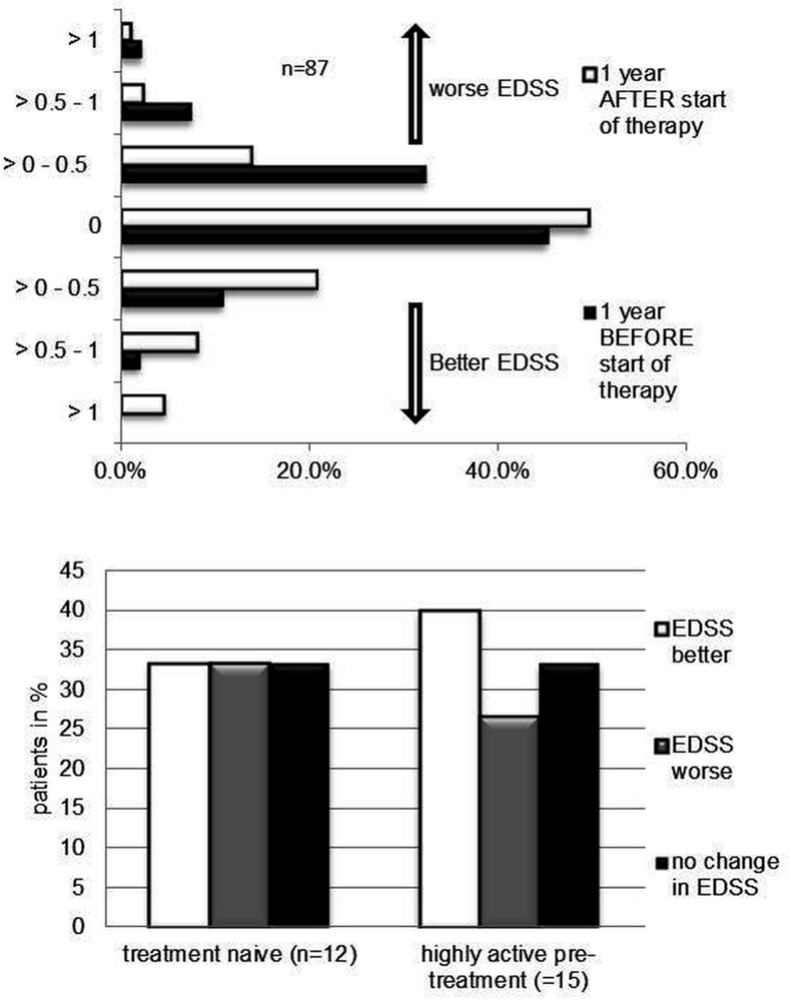
A Proportion of patients in percent with improving, worsening or stable EDSS during the year before (black columns) and after the first year (white columns) of fingolimod treatment. B. Proportion of patients in percent with improving (white columns), worsening (grey columns) or stable EDSS (black columns) during the first year of fingolimod treatment in a treatment naïve patient group compared to patients previously treated with a highly active treatment.

To assess the disease evolution the EDSS was determined one year before start of fingolimod, at baseline and after one year of fingolimod treatment. There was a significant increase in the percentage of patients whose EDSS improved by at least 0.5 points or remained stable in the first year of fingolimod treatment as compared to the year before initiation of therapy (patients stable or improved: 82.7% under fingolimod vs. 58.1% before fingolimod; p<0.01; Fig 1A). Due to the limited follow up of one year data for calculation of confirmed disability progression were not available.

**Fig 2:**
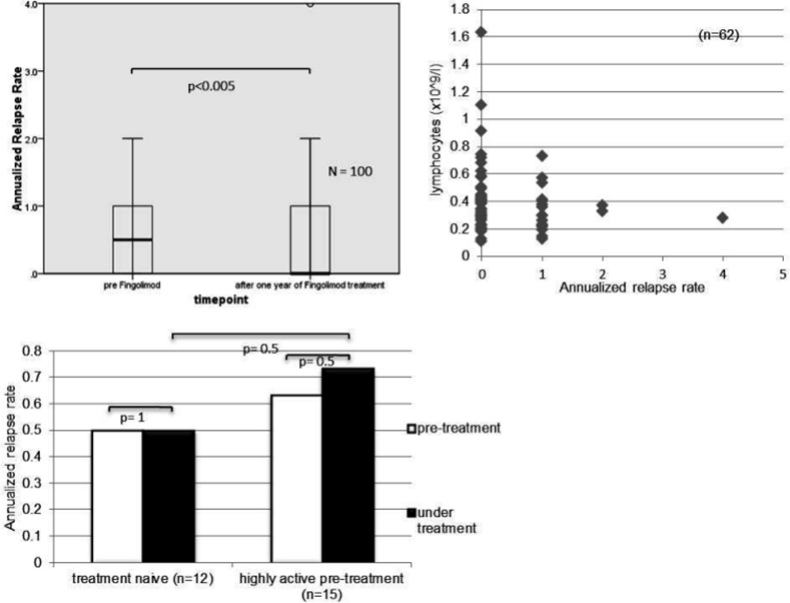
A. Annualized relapse, rate (ARR) during the first year of fingolimod treatment compared to the year before fingolimod initiation. B. Correlation between ARR and the lymphocyte levels during the observation period. C. ARR during the first year of fingolimod treatment (black column) compared to the year before fingolimod initiation (white column) in treatment naïve and highly active treated patients.

In line with the improvement in EDSS there was an overall reduction in relapse rate during the first year of fingolimod treatment compared to the previous year. The median of the annualized relapse rate (ARR) dropped from 0.5 to 0 (p<0.005; Fig 2A). There was no correlation between ARR and blood lymphocyte levels (correlation coefficient -0.15, p = 0.23; Fig 2B).

**Fig 3:**
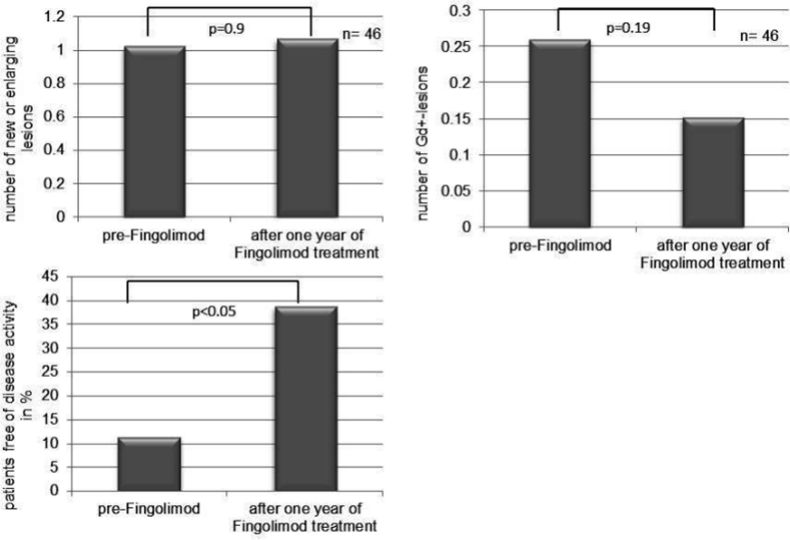
Comparison of MRI parameters at baseline and after one year of fingolimod therapy. **A.** Number of new or enlarging T2 lesions. **B.** Number of Gd+-lesions. **C.** Proportion of patients with NEDA in the year before fingolimod start and after one year of fingolimod treatment.

In 46 patients of our cohort, comparable MRIs were available to assess lesion development during the first year of fingolimod treatment compared to the previous year. MRI activity was defined as new or enlarging lesions on T2-weighted images or Gadolinium (Gd)-lesions on T1-weighted images. The number of new lesions remained stable throughout the observation period (mean of 1.02 (standard error of the mean, SEM, 0.28) and 1.07 (SEM 0.28) new lesions per scan during the year before fingolimod treatment and in the first year of treatment respectively; p = 0.9; Fig 3A). Gd-enhancing lesions dropped from a mean of 0.26 (SEM 0.09) lesions per scan to a mean of 0.15 (SEM 0.05, p = 0.19; Fig 3B).

In the same subgroup of patients we determined the number of patients with no evidence of disease activity (NEDA), i.e. patients without progression in the EDSS, no relapses, no new or enlarging T2 lesions, and no Gd-enhancing lesions on MRI. Whereas before fingolimod treatment only 10% of these patients qualified for NEDA, this number increased to 37% (p< 0.05; Fig 3C).

To investigate the influence of pretreatment on the efficacy of fingolimod we analyzed 15 patients with high activity and treatment with either natalizumab or mitoxantrone prior to fingolimod initiation and compared them to a group of 12 patients with no immunomodulatory treatment in the two previous years. The group of MS patients with active pre-treatment had a higher baseline EDSS compared to the patients without previous treatment (EDSS 4.2 versus 1.9; p<0.01), but a similar disease duration of 9 years (p = 0.9). There was no significant difference in the EDSS (p = 0.4 for the patients with highly active pre-treatment; p = 0.7 for the patients without previous treatment) and ARR (p = 0.5 for the patients with highly active pre-treatment; p = 1 for the patients without previous treatment) between the year before and the year on fingolimod therapy in these two groups (Figs 1B and 2C), neither did we observe a significant difference comparing the ARR of treatment naïve and highly active patients in the first year of fingolimod treatment (p = 0.5).

**Fig 4:**
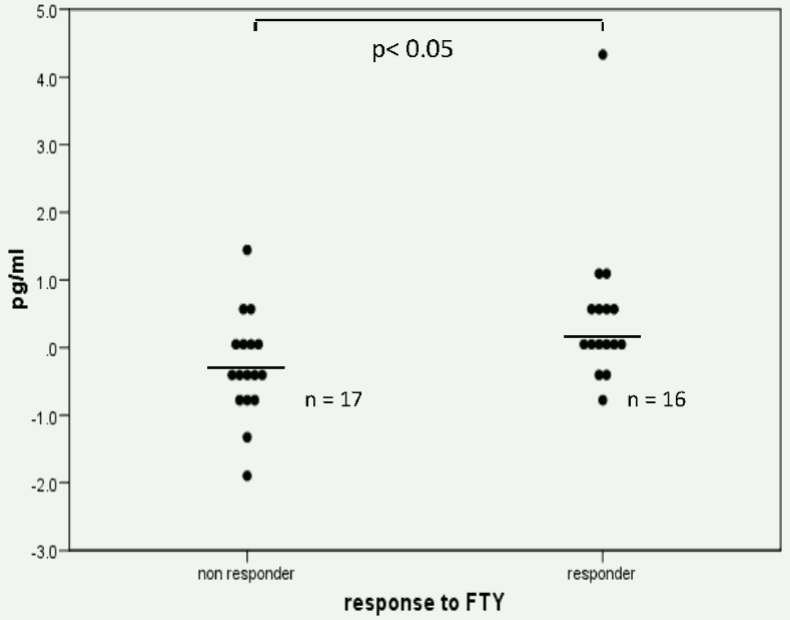
Mean difference of IFNgamma expression between baseline and three months under fingolimod treatment in responders and non-responders.

To identify potential predictive biomarkers the clinical outcomes were correlated to changes in serum cytokines (GM-CSF, IFNgamma, TNFa, IL-1b, IL-2, IL-6, IL-8, IL-10, and IL-12p70) at start of therapy and three months after initiation of fingolimod in a subgroup of 33 patients. We defined patients as being responders when there was neither clinical nor MRI activity (Gd-enhancing lesions or new or enlarging T2 lesions) during the first year of fingolimod treatment and as non-responders when there was either clinical disease and/or MRI activity. None of these patients received a corticosteroid treatment in the three months before blood sampling. We found a significant increase of IFNgamma (IFNg, p< 0.05) in responders versus non-responders (Fig 4). The increase of IFNgamma in the responder group remained significant even after excluding one outlier (IFNgamma: 4.3pg/ml). After correction for multiple testing the result did not remain significant (p = 0.23). The other seven cytokines showed fluctuations with no real trend and no difference between responders and non-responders.

## Discussion

Evidence from three phase III trials demonstrated efficacy of fingolimod in patients with RR-MS. However, this evidence is limited to a selected patient population. This raises questions regarding the efficacy and safety of fingolimod in a real-world patient population. In 2012 several reports about cardiac events and sudden unexplained deaths in the context of fingolimod brought up concerns about the cardiovascular risk profile of fingolimod [5] [6, 7]. Our cohort included 10% of patients with cardiac risk factors. We recorded an incidence rate of 4% for AV-block I° and 1% for AV-block II°. All patients were asymptomatic. Our incidence rates for AV-blocks were higher compared to those observed in the phase III trial FREEDOMS, but are in line with a recent trial assessing cardiac safety of fingolimod, the FIRST trial [8], which showed an incidence of 1.2% for AV-block II°. Compared to the FIRST study our cohort had a follow-up of one year, during which no novel cardiac events emerged.

The second safety concern of fingolimod is a potentially increased risk of viral infections. During phase III clinical trials a fatal primary VZV infection and a HSV-1 encephalitis have been reported [9]. Several recent case reports indicated that fingolimod might raise the risk for VZV infection [10]. In our cohort we observed 3 patients with zoster during one year which is around 5 times higher than it is expected for the general population. We previously showed that the cellular immune response against VZV is compromised in fingolimod treated patients [11]. These data suggest that there might be a slightly increased risk of VZV reactivation in fingolimod treated patients.

The efficacy analysis of fingolimod in our cohort showed a significant relapse rate reduction compared to the year before fingolimod. No evidence of disease activity (NEDA) was reached by 39% of our patients during the observation period. This compares to 33% fulfilling NEDA criteria in the FREEDOMS trial during a two year observation period. This similarity between clinical trial and clinical practice is not self-evident because the patients in our cohort were more advanced in their disease. The patients in our cohort were older (44 versus 37 years), had a longer disease duration (13 versus 8 years), and a more advanced EDSS (3.1 versus 2.4). Only 12% were treatment-naïve before fingolimod start compared to 57% in the FREEDOMS trial. Although all these parameters are thought to be negative predictive factors for a beneficial therapeutic response, we still could detect a favourable response to fingolimod [12]. However, when considering efficacy results of this cohort the observational open label nature of this study has to be kept in mind along withthe number of patients and the limited follow-up time.

Comparing serum cytokines there was a significant increase of IFNg in responders versus non-responders. At first glance this seems contradictory since INFg has been described as a proinflammatory Th1 cytokine, inducing CNS inflammation in animal models [13, 14] and leading to an MS exacerbation in a clinical trial [15]. On the other hand there are reports that mice deficient for the IFNg receptor are more susceptible for and develop a more severe EAE [16] and that EAE disease severity is inhibited by induction of IFNg early during disease course [17]. Considering the limited sample size it remains to be shown if this increase of IFNg in responders can be reproduced in a larger prospective cohort of patients. Since this result did not remain significant after correcting for multiple testing it has to be interpreted with caution.

In summary, this study in a real-world setting adds data primarily on safety of fingolimod in an unselected patient population that differs significantly from the population that has been investigated in clinical trials. Data on efficacy are to be seen with caution because of the lack of a control group, the lack of blinding, and the limited follow-up. Despite these limitations it is conceivable that many patients profit from a switch to fingolimod.

## References

[1] Mehling M, Kappos L, Derfuss T. Fingolimod for multiple sclerosis: mechanism of action, clinical outcomes, and future directions. Current neurology and neuroscience reports. 2011;11(5):492–7. Epub 2011/07/27. 10.1007/s11910-011-0216-9 . 21789537

[2] Kappos L, Radue E-W, O'Connor P, Polman C, Hohlfeld R, Calabresi P, et al A Placebo-Controlled Trial of Oral Fingolimod in Relapsing Multiple Sclerosis. New England Journal of Medicine. 2010;362(5):387–401. 10.1056/NEJMoa0909494 . 20089952

[3] Cohen JA, Barkhof F, Comi G, Hartung H-P, Khatri BO, Montalban X, et al Oral Fingolimod or Intramuscular Interferon for Relapsing Multiple Sclerosis. New England Journal of Medicine. 2010;362(5):402–15. 10.1056/NEJMoa0907839 . 20089954

[4] Martins TB, Rose JW, Jaskowski TD, Wilson AR, Husebye D, Seraj HS, et al Analysis of proinflammatory and anti-inflammatory cytokine serum concentrations in patients with multiple sclerosis by using a multiplexed immunoassay. American journal of clinical pathology. 2011;136(5):696–704. Epub 2011/10/28. 10.1309/AJCP7UBK8IBVMVNR . 22031307

[5] Lindsey JW, Haden-Pinneri K, Memon NB, Buja LM. Sudden unexpected death on fingolimod. Multiple sclerosis. 2012;18(10):1507–8. Epub 2012/02/04. 10.1177/1352458512438456 . 22300970

[6] Faber H, Fischer HJ, Weber F. Prolonged and symptomatic bradycardia following a single dose of fingolimod. Multiple sclerosis. 2013;19(1):126–8. Epub 2012/06/26. 10.1177/1352458512447596 . 22729989

[7] Espinosa PS, Berger JR. Delayed fingolimod-associated asystole. Multiple sclerosis. 2011;17(11):1387–9. Epub 2011/06/10. 10.1177/1352458511410344 . 21652609

[8] Gold R, Comi G, Palace J, Siever A, Gottschalk R, Bijarnia M, et al Assessment of cardiac safety during fingolimod treatment initiation in a real-world relapsing multiple sclerosis population: a phase 3b, open-label study. Journal of neurology. 2013 Epub 2013/11/14. 10.1007/s00415-013-7115-8 . 24221641 PMC3915082

[9] Uccelli A, Ginocchio F, Mancardi GL, Bassetti M. Primary varicella zoster infection associated with fingolimod treatment. Neurology. 2011;76(11):1023–4. Epub 2011/03/16. 10.1212/WNL.0b013e31821043b5 . 21403115

[10] Ratchford JN, Costello K, Reich DS, Calabresi PA. Varicella-zoster virus encephalitis and vasculopathy in a patient treated with fingolimod. Neurology. 2012;79(19):2002–4. Epub 2012/10/05. 10.1212/WNL.0b013e3182735d00 23035072 PMC3484989

[11] Ricklin ME, Lorscheider J, Waschbisch A, Paroz C, Mehta SK, Pierson DL, et al T-cell response against varicella-zoster virus in fingolimod-treated MS patients. Neurology. 2013;81(2):174–81. Epub 2013/05/24. 10.1212/WNL.0b013e31829a3311 . 23700335

[12] Nixon R, Eckert B, Cutter G, Mercier F, Francis G, Kappos L. Indirect comparisons of oral fingolimod versus natalizumab and cladribine for the treatment of relapsing–remitting multiple sclerosis based on data from FREEDOMS, AFFIRM and CLARITY. ENS: 22nd meeting of the European Neurological Society. 2012.

[13] Sethna MP, Lampson LA. Immune modulation within the brain: recruitment of inflammatory cells and increased major histocompatibility antigen expression following intracerebral injection of interferon-gamma. Journal of neuroimmunology. 1991;34(2–3):121–32. . 1918319 10.1016/0165-5728(91)90121-m

[14] Suryani S, Sutton I. An interferon-gamma-producing Th1 subset is the major source of IL-17 in experimental autoimmune encephalitis. Journal of neuroimmunology. 2007;183(1–2):96–103. 10.1016/j.jneuroim.2006.11.023 . 17240458

[15] Panitch HS, Hirsch RL, Haley AS, Johnson KP. Exacerbations of multiple sclerosis in patients treated with gamma interferon. Lancet. 1987;1(8538):893–5. . 2882294 10.1016/s0140-6736(87)92863-7

[16] Willenborg DO, Fordham S, Bernard CC, Cowden WB, Ramshaw IA. IFN-gamma plays a critical down-regulatory role in the induction and effector phase of myelin oligodendrocyte glycoprotein-induced autoimmune encephalomyelitis. Journal of immunology. 1996;157(8):3223–7. . 8871615

[17] Gran B, Chu N, Zhang GX, Yu S, Li Y, Chen XH, et al Early administration of IL-12 suppresses EAE through induction of interferon-gamma. Journal of neuroimmunology. 2004;156(1–2):123–31. 10.1016/j.jneuroim.2004.07.019 . 15465603

